# Application of Least Squares with Conditional Equations Method for Railway Track Inventory Using GNSS Observations

**DOI:** 10.3390/s20174948

**Published:** 2020-09-01

**Authors:** Krzysztof Czaplewski, Zbigniew Wisniewski, Cezary Specht, Andrzej Wilk, Wladyslaw Koc, Krzysztof Karwowski, Jacek Skibicki, Paweł Dabrowski, Bartosz Czaplewski, Mariusz Specht, Piotr Chrostowski, Jacek Szmaglinski, Slawomir Judek, Slawomir Grulkowski, Roksana Licow

**Affiliations:** 1 Department of Geodesy and Oceanography, Faculty of Navigation, Gdynia Maritime University, 81-374 Gdynia, Poland; c.specht@wn.umg.edu.pl (C.S.); p.dabrowski@wn.umg.edu.pl (P.D.); 2 Department of Geodesy, Faculty of Geoengineering, University of Warmia and Mazury, 10-719 Olsztyn, Poland; zbyszekw@uwm.edu.pl; 3 Department of Electrified Transportation, Faculty of Electrical and Control Engineering, Gdansk University of Technology, 80-233 Gdansk, Poland; andrzej.wilk@pg.edu.pl (A.W.); wladyslaw.koc@pg.edu.pl (W.K.); krzysztof.karwowski@pg.edu.pl (K.K.); jacek.skibicki@pg.edu.pl (J.S.); slawomir.judek@pg.edu.pl (S.J.); 4 Department of Teleinformation Networks, Faculty of Electronics, Telecommunications and Informatics, Gdansk University of Technology, 80-233 Gdansk, Poland; bartosz.czaplewski@eti.pg.edu.pl; 5 Department of Transport and Logistics, Faculty of Navigation, Gdynia Maritime University, 81-225 Gdynia, Poland; m.specht@wn.umg.edu.pl; 6 Department of Rail Transportation and Bridges, Faculty of Civil and Environmental Engineering, Gdansk University of Technology, 80-233 Gdansk, Poland; piotr.chrostowski@pg.edu.pl (P.C.); jacek.szmaglinski@pg.edu.pl (J.S.); slawomir.grulkowski@pg.edu.pl (S.G.); roksana.licow@pg.edu.pl (R.L.)

**Keywords:** GNSS measurements, estimation methods, rail transport, railway infrastructure

## Abstract

Satellite geodetic networks are commonly used in surveying tasks, but they can also be used in mobile surveys. Mobile satellite surveys can be used for trackage inventory, diagnostics and design. The combination of modern technological solutions with the adaptation of research methods known in other fields of science offers an opportunity to acquire highly accurate solutions for railway track inventory. This article presents the effects of work carried out using a mobile surveying platform on which Global Navigation Satellite System (GNSS) receivers were mounted. The satellite observations (surveys) obtained were aligned using one of the methods known from classical land surveying. The records obtained during the surveying campaign on a 246th km railway track section were subjected to alignment. This article provides a description of the surveying campaign necessary to obtain measurement data and a theoretical description of the method employed to align observation results as well as their visualisation.

## 1. Introduction

The development of modern rail transport is associated, inter alia, with increasing train speed. This causes specific problems related to railway traffic control, as described, for example, in [1], or with designing fast railway stops as described, for example, in [2]. An increase in train speed requires, primarily, that a high level of rail transport safety should be ensured, which is not possible without well-developed railway infrastructure constructed with the utmost care. Therefore, there is a need to monitor trackages, both at the construction stage and during operation [3]. The assessment of railway track is important not only for assurance of a high level of rail transport, but it is also necessary for providing high-quality transport services, as described in [4]. For the assessment of railway track geometry, track gauges and measuring trolleys are used, whose application examples can be found in the literature on the subject, e.g., in [5] or in [6]. However, in the last twenty years, Global Navigation Satellite Systems (GNSS) have become the very basic precise instrument used worldwide. GNSS and the relative instrumentation are under systematic development and constantly increase the application possibilities, which has resulted in their widespread use despite the emerging hazards in operation [7,8]. The main application of GNSS is in geodesy but they are more often used to determine the position of objects in motion [9,10] in the process of directly ensuring a high level of safety of maritime navigation, e.g., [11], or they support the process of modelling the surrounding reality in the computer environment, e.g., [12]. It has become clear that there are possibilities for the application of GNSS in railway transport, e.g., for monitoring railway infrastructure.

The proposal for the performance of mobile surveys involving the geodetic inventory of railway tracks using Global Navigation Satellite Systems (GNSS) in railway engineering appeared for the first time in Poland [13]. The proposals for the new use of positioning techniques were verified during the initial experimental tests while taking an inventory of the railway route between Koscierzyna and Kartuzy in Poland [14], when four GNSS receivers were used as well as the ASG-EUPOS Polish active satellite GNSS surveying network [15]. The study results confirmed the extensive application possibilities for the method and drew attention to its limitations due to signal availability in a built-up area. Launching new GNSS systems (Galileo, Beidou) and creating multiconstellation GNSS surveying networks [16] while increasing the accuracy of the existing ones (Navstar GPS/Glonass) allowed the authors to develop this method [17]. In the period 2009–2017, while developing the method of mobile satellite surveys, research was carried out in two main directions, i.e., the land surveying method associated with increasing accuracy and availability of measurements ([18,19,20]) and the construction method aimed at developing new design and operational methods [21,22,23,24,25]. At other research centres, analogous studies were also undertaken to integrate tacheometric and GNSS surveys [26], with measurement support through the application of inertial navigation systems (INS) [27,28] or the application of mobile laser scanners [29,30]. In addition, studies were conducted to apply multisensor methods [31,32,33,34,35,36].

A very important aspect is increasing the accuracy of determining the position of designated vehicles using GNSS. To this end, it is worth applying techniques and methods for aligning observation results used in land surveying. The literature provides examples of the application of land surveying alignment methods in rail transport, e.g., [37] which described the application of the conditional observation alignment method or referred to the estimation of the accuracies obtained during traditional static surveys [38]. In [38] the authors describe the use of automated measurement systems based on trolleys for track surveying supported with automatic tacheometric surveys. Moreover, there are examples of the application of geodetic alignment methods in maritime transport, e.g., [39,40]. These articles present the results of work related to the adaptation of a surveying method for aligning observation results, known as the method of least squares with conditional equations in mobile surveys. The alignment process was checked using the records of GNSS signals during the mobile surveying campaign conducted in July 2019 on a 246-km-long section between Tczew–Chojnice–Brusy–Chojnice–Tczew located in northern Poland.

## 2. Mobile Surveying Platform

**Figure 1:**
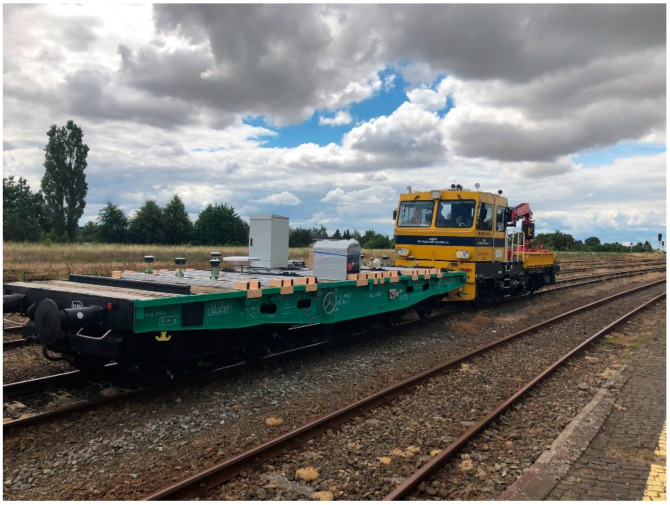
Mobile surveying platform.

To obtain GNSS surveying data that enable an assessment of the railway track geometry during dynamic surveys (in motion), it was necessary to design and build a mobile surveying platform (MSP). The MSP was conceptualised and developed as part of a research project implemented by the Gdansk University of Technology and Gdynia Maritime University. The mobile surveying platform comprises a railway car (Figure 1) on which nine mounted guides have been installed to mount measuring instruments (GNSS receivers, INS systems, a mobile laser scanner and visual systems), a power generator and a workstation recording data from the installed sensors.

On the surveying platform prepared in this way, any given number of measuring instruments can be installed. During the surveys, the car was connected to a DH-350.11 hydraulic handcar. In this configuration, the test team can configure recorders depending on the type of research for which it is to be used.

## 3. Theoretical Basis of the Method of Least Squares with Conditional Equations

In geodetic surveys, there are alignment tasks in which the parameters of the system of observation equations must satisfy additional conditions [41]. In geodetic networks, these conditions may concern the coordinates of certain points being part of a particular network. Such a case occurs when the distance between points is determined with high accuracy, or the conditions between the coordinates result from the geometric structure of the network. Such a case is found in the research problem under consideration, where the position of antennas on the surveying platform are determined from satellite observations as well as from tacheometric surveys conducted prior to the measurement session using satellite measurement techniques. The distances between GNSS receiver antennas were precisely measured prior to commencing dynamic surveys. The geodetic inventory of GNSS antennas was carried out using an electronic total station and the values obtained were adopted as observations determined with high accuracy, which justifies the assumption that they are relatively error-free. Therefore, it follows that the parameters of the observation system (coordinates of GNSS antennas’ measuring points on the surveying platform) must satisfy additional conditions, i.e., the distances between GNSS antennas on the MSP. For the cumulative processing of the obtained observations, the Method of Least Squares with conditional equations (LSce) can then be applied. The theory behind the method was discussed in detail inter alia in [42,43]. Its further developments and applications to prepare geodetic observations are discussed inter alia in [41], [44,45]. It should also be noted that conditional equations have also been used in other estimation methods, e.g., in M_split_ estimation [46]. The initial tests of the application of LSce to estimate the GNSS receiver antennas’ coordinates on surveying platforms were carried out over the test section of tram tracks in the urban agglomeration of Gdansk [47].

Below are the main assumptions and the ways to apply LSce in the context of the surveying platform and the associated observation systems considered in this study.

Let us assume that the coordinates of antennas $\left(x_{i},y_{i}\right)$, i=1,2,⋯,s, on the surveying platform, are contained in the vector $X={\left[x_{1},y_{1},\ldots ,x_{s},y_{s}\right]}^{T}={\left[X_{1},X_{2},\ldots ,X_{r}\right]}^{T}$ (*s*—the number of antennas, *r*—the number of unknown coordinates). Following the previous assumptions about the adopted measurement structure, these quantities must satisfy the following system of conditional equations:

(1) $$\begin{array}{l} \Psi_{1}\left(X_{1},X_{2},\ldots ,X_{r}\right)=0\\ \Psi_{2}\left(X_{1},X_{2},\ldots ,X_{r}\right)=0\\ \vdots \\ \Psi_{w}\left(X_{1},X_{2},\ldots ,X_{r}\right)=0 \end{array}\}\;\Leftrightarrow \Psi \left(X\right)=0$$

For example, if we assume that $\left(x_{i},y_{i}\right)$ $\left(x_{j},y_{j}\right)$ are the coordinates of two antennas between which the distance $d_{ij}$ was measured with high accuracy, then ${\left[{\left(x_{j}-x_{i}\right)}^{2}+{\left(y_{j}-y_{i}\right)}^{2}\right]}^{1/2}-d_{ij}=0$. Moreover, in accordance with the general principles of determining geodetic network coordinates, the vector X is determined based on GNSS satellite observations which enable the formulation of the following system of observation equations:

(2) $$\begin{array}{l} y_{1}+v_{1}=F_{1}\left(X_{1},X_{2},\ldots ,X_{r}\right)\\ y_{2}+v_{2}=F_{2}\left(X_{1},X_{2},\ldots ,X_{r}\right)\\ \vdots \\ y_{n}+v_{n}=F_{n}\left(X_{1},X_{2},\ldots ,X_{r}\right) \end{array}\}\;\Leftrightarrow y+v=F\left(X\right)$$

where $y={\left[y_{1},y_{2}\cdots ,y_{n}\right]}^{T}$ is the observation vector. Using $v={\left[v_{1},v_{2}\cdots ,v_{n}\right]}^{T}$, the vector of random observation errors with the covariance matrix $C_{v}=\sigma_{0}^{2}Q=\sigma_{0}^{2}P_{}^{-1}$ and the vector of expected values E(v)=0 (Q—known cofactor matrix, P—weight matrix, $\sigma_{0}^{2}$—unknown variance coefficient). When we assume that the approximate coordinates $X^{0}={\left[X_{1}^{0},\ldots ,X_{r}^{0}\right]}^{T}$ of GNSS receiver antennas are known, then the equation y+v=F(X) can be replaced with a linear observation equation with the following form:

(3) $$v=F\left(X\right)-y=F\left(X^{0}+dX\right)-y={\frac{\partial F\left(X\right)}{\partial X}|}_{X=X^{0}}dX+F\left(X^{0}\right)-y=AdX+l$$

The quantity dX is the vector of unknown coordinate increments, such that $X=X^{0}+dX$. The matrix

$$A={\frac{\partial F\left(X\right)}{\partial X}|}_{X=X^{0}}={\left[\begin{array}{cccc} \frac{\partial F_{1}\left(X\right)}{\partial X_{1}} & \frac{\partial F_{1}\left(X\right)}{\partial X_{2}} & \cdots & \frac{\partial F_{1}\left(X\right)}{\partial X_{r}}\\ \frac{\partial F_{2}\left(X\right)}{\partial X_{1}} & \frac{\partial F_{2}\left(X\right)}{\partial X_{2}} & \cdots & \frac{\partial F_{2}\left(X\right)}{\partial X_{r}}\\ \cdots & \cdots & \cdots & \cdots \\ \frac{\partial F_{n}\left(X\right)}{\partial X_{1}} & \frac{\partial F_{n}\left(X\right)}{\partial X_{2}} & \cdots & \frac{\partial F_{n}\left(X\right)}{\partial X_{r}} \end{array}\right]}_{X=X^{0}}$$

is the design of the system of linear equations, while

$$l=F\left(X^{0}\right)-y={\left[F_{1}\left(X^{0}\right)-y_{1},\;F_{2}\left(X^{0}\right)-y_{2},\;\cdots ,\;F_{n}\left(X^{0}\right)-y_{n}\right]}^{T}$$

is the absolute term vector. Let us also assume that the matrix A is a column full rank, i.e., rank(A)=r. Considering that $X=X^{0}+dX$, the system of observation equations (1) can also be similarly brought to the linear form. After developing the function Ψ(X) to the form

(4) $$\Psi \left(X\right)=\Psi \left(X^{0}+dX\right)=\Psi \left(X^{0}\right)+{\frac{\partial \Psi \left(X\right)}{\partial X}|}_{X=X^{0}}dX=\Psi \left(X^{0}\right)+BdX$$

the following linear conditional equation is obtained:

(5) BdX+Δ=0

where:

$$B={\frac{\partial \Psi \left(X\right)}{\partial X}|}_{X=X^{0}}={\left[\begin{array}{cccc} \frac{\partial \Psi_{1}\left(X\right)}{\partial X_{1}} & \frac{\partial \Psi_{1}\left(X\right)}{\partial X_{2}} & \cdots & \frac{\partial \Psi_{1}\left(X\right)}{\partial X_{r}}\\ \frac{\partial \Psi_{2}\left(X\right)}{\partial X_{1}} & \frac{\partial \Psi_{2}\left(X\right)}{\partial X_{2}} & \cdots & \frac{\partial \Psi_{2}\left(X\right)}{\partial X_{r}}\\ \cdots & \cdots & \cdots & \cdots \\ \frac{\partial \Psi_{w}\left(X\right)}{\partial X_{1}} & \frac{\partial \Psi_{w}\left(X\right)}{\partial X_{2}} & \cdots & \frac{\partial \Psi_{w}\left(X\right)}{\partial X_{r}} \end{array}\right]}_{X=X^{0}},$$

$$\Delta =\Psi \left(X^{0}\right)={\left[\Psi_{1}\left(X^{0}\right),\;\Psi_{2}\left(X^{0}\right),\;\cdots ,\;\Psi_{w}\left(X^{0}\right)\right]}^{T}.$$

Taking into account the conditions binding the parameters being determined, and the application of the objective function of the least-squares method, it leads to the optimisation of the problem with the following form (e.g., [38]):

(6) $$\begin{array}{l} AdX+l=v\\ BdX+\Delta =0\\ C_{v}=\sigma_{0}^{2}P_{}^{-1}\\ \varphi (dX)=v^{T}Pv=\min \end{array}\}$$

In order to exactly solve this problem, it is necessary to replace the original objective function $\varphi (dX)=v^{T}Pv$ with the Lagrange function with the following form (e.g., [39])

(7) $$\varphi_{L}(dX)=\varphi (dX)-2\kappa^{T}(BdX+\Delta )=v^{T}Pv-2\kappa^{T}(BdX+\Delta )$$

where κ is the Lagrange’s unknown multiplier vector. The optimisation problem $\min\varphi_{L}(dX)=\varphi_{L}(d\hat{X})$ solves such quantities for which the following sufficient conditions are satisfied:

(8) $${\frac{\partial \varphi_{L}(dX)}{\partial dX}|}_{\hat{\kappa },d\hat{X}}={\frac{\partial \varphi_{L}(dX)}{\partial v}\frac{\partial v}{\partial dX}|}_{\hat{\kappa },d\hat{X}}=2{\hat{v}}^{T}PA-2{\hat{\kappa }}^{T}B=0$$

(9) $${\frac{\partial \varphi_{L}(dX)}{\partial \hat{\kappa }}|}_{\hat{\kappa },d\hat{X}}=Bd\hat{X}+\Delta =0$$

Moreover, in the issue under consideration, the necessary condition involving the positive-definite of the second derivative $\partial^{2}\varphi_{L}(dX)/\partial v\partial v^{T}=2P$ should be satisfied here. It follows from Equation (8) that

(10) $$AP\hat{v}-B^{T}\hat{\kappa }=0\;\Leftrightarrow \;A^{T}PAd\hat{X}+A^{T}Pl-B^{T}\hat{\kappa }=0$$

If $rank(A^{T}PA)=rank(A)=r$, then the solution to Equation (10) is vector

(11) $$d\hat{X}=-{\left(A^{T}PA\right)}^{-1}(A^{T}Pl-B^{T}\hat{\kappa })$$

The correlate vector must satisfy the condition (9). By substituting vector $d\hat{X}$ described with the dependence (11) to the equation $Bd\hat{X}+\Delta =0$, the following is obtained:

(12) $$\hat{\kappa }=-{\left[B{\left(A^{T}PA\right)}^{-1}B^{T}\right]}^{-1}\left[\Delta -B{\left(A^{T}PA\right)}^{-1}A^{T}PL\right]$$

In practice, task (6) can also be solved in a different way that is simpler from the numerical perspective. To this end, the conditional equation BdX+Δ=0 should be replaced with the equivalent observation equation:

(13) $$BdX+\Delta =v_{*}$$

Since it is required that $v_{*}=0$, therefore the vector of fictitious observation errors $v_{*}$ should be assigned such a covariance matrix $C_{v_{*}}=\sigma_{0}^{2}P_{*}^{-1}$ so that also fictitious corrections ${\hat{v}}_{*}$ meet the condition ${\hat{v}}_{*}=0$ (within the calculation precision limits). Thus, task (6) is replaced with the optimisation problem, classical for the LS-method, with the following form:

(14) $$\begin{array}{l} \begin{array}{c} AdX+l=v\\ \begin{array}{l} BdX+\Delta =v_{*}\\ C_{v}=\sigma_{0}^{2}P_{}^{-1}\\ C_{v_{*}}=\sigma_{0}^{2}P_{*}^{-1} \end{array} \end{array}\\ \varphi (dX)=v^{T}Pv+v_{*}^{T}P_{*}v_{*}=\min \end{array}\}\;\Leftrightarrow \;\left\{ \begin{array}{l} \overset{⌢}{A}dX+\overset{⌢}{l}=\overset{⌢}{v}\\ {\overset{⌢}{C}}_{v}=\sigma_{0}^{2}{\overset{⌢}{P}}_{}^{-1}\\ \varphi (dX)={\overset{⌢}{v}}^{T}\overset{⌢}{P}\overset{⌢}{v}=\min \end{array}\right.$$

where:

$$\overset{⌢}{A}=\left[\begin{array}{c} A\\ B \end{array}\right],\;\overset{⌢}{l}=\left[\begin{array}{c} l\\ \Delta \end{array}\right],\;\overset{⌢}{v}=\left[\begin{array}{l} v\\ v_{*} \end{array}\right].$$

Vector $\overset{⌢}{v}$ is the total vector of observation errors with the covariance matrix of:

(15) $$\overset{⌢}{C}=\left[\begin{array}{cc} C_{v}=\sigma_{0}^{2}P_{}^{-1} & 0\\ 0 & C_{v_{*}}=\sigma_{0}^{2}P_{*}^{-1} \end{array}\right]=\sigma_{0}^{2}\left[\begin{array}{cc} P_{}^{-1} & 0\\ 0 & P_{*}^{-1} \end{array}\right]=\sigma_{0}^{2}{\overset{⌢}{P}}^{-1}$$

where $\overset{⌢}{P}=\mathrm{diag}(P,\;P_{*})$ is the total weight matrix ($\sigma_{0}^{2}$—variance coefficient common for both components of the model (15). The solution to task (14) is the increment estimator $d\hat{X}$ with the following form:

(16) $$d\hat{X}=-{\left({\overset{⌢}{A}}^{T}\overset{⌢}{P}\overset{⌢}{A}\right)}^{-1}{\overset{⌢}{A}}^{T}\overset{⌢}{P}\overset{⌢}{l}$$

where: ${\overset{⌢}{A}}^{T}\overset{⌢}{P}\overset{⌢}{A}=A^{T}PA+B^{T}P_{*}B$, ${\overset{⌢}{A}}^{T}\overset{⌢}{P}\overset{⌢}{l}=A^{T}Pl+B^{T}P_{*}\Delta$. By determining the covariance matrix for this estimator, the following is obtained:

(17) $$C_{d\hat{X}}={\hat{\sigma }}_{0}^{2}{\left({\overset{⌢}{A}}^{T}\overset{⌢}{P}\overset{⌢}{A}\right)}^{-1}$$

where:

(18) $${\hat{\sigma }}_{0}^{2}=\frac{{\hat{\overset{⌢}{v}}}^{T}\overset{⌢}{P}\hat{\overset{⌢}{v}}}{n+w-r}$$

is an estimator of variance coefficient $\sigma_{0}^{2}$. The vector $\hat{\overset{⌢}{v}}={\left[{\hat{v}}_{}^{T},\;{\hat{v}}_{*}^{T}\right]}^{T}$ is a total correction vector determined from the following dependence:

(19) $$\hat{\overset{⌢}{v}}=\overset{⌢}{A}d\hat{X}+\overset{⌢}{l}$$

The estimator of the GNSS receiver antennas’ coordinates is the vector:

(20) $$\hat{X}=X^{0}+d\hat{X}$$

with the covariance matrix $C_{\hat{X}}=C_{d\hat{X}}$ It should be noted that the diagonal elements of this matrix are the squares of mean errors of the determined estimators, i.e., $m_{{\hat{X}}_{i}}^{2}={\left[C_{\hat{X}}\right]}_{ii}$.

Considering both presented solutions, the exact one represented by the alignment task (6) with the estimator (11), and the equivalent one represented by the alignment task (14) with the estimator (16) yield the same solutions. Therefore, the equivalent solution that was used in the problem described in this article is more often applied in technical applications.

## 4. Practical Applications of GNSS in Railway Surveys and Alignment Results

**Figure 2:**
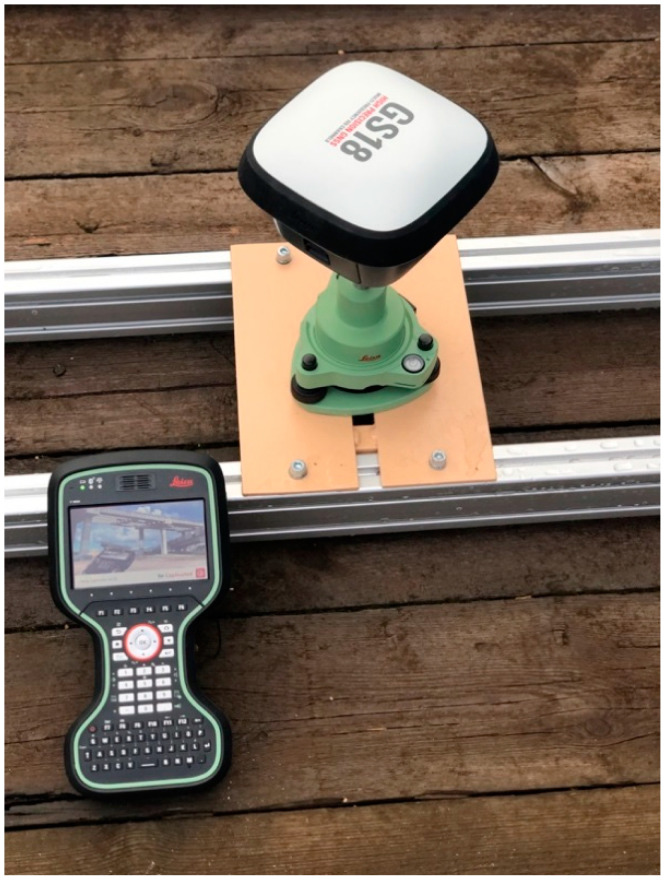
A LEICA GS-18 receiver used during the surveys.

On 16–17 July 2019, on a route between Tczew–Chojnice–Brusy–Chojnice–Tczew, located in northern Poland, a study was conducted using six Leica GS-18 GNSS receivers (Figure 2).

Selected technical data of the receiver are provided in the Table 1.

**Figure 3:**
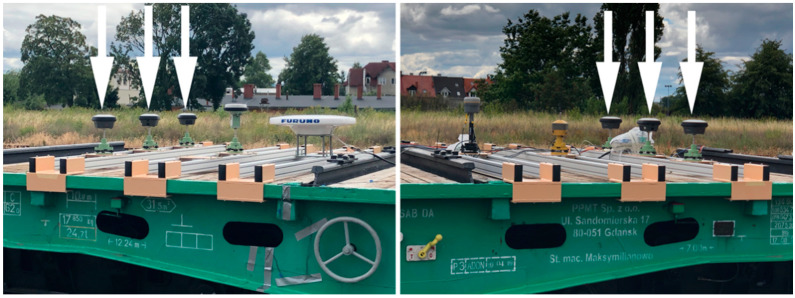
Global Navigation Satellite System (GNSS) receivers by Leica mounted on the mobile surveying platform.

On the day preceding the proper measurements, surveying devices and instruments were mounted on a railway car (Figure 3).

**Figure 4:**
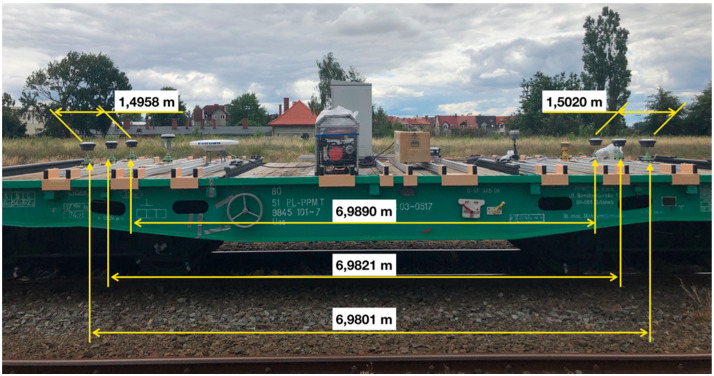
Determined distances between antennas on the surveying platform.

To precisely determine the distance between the receiver antennas axes, tacheometric surveys were then conducted using a LEICA TS 1103 total station. The measured distances are provided in Figure 4.

Six GNSS receivers were used in the study. Furthermore, 507,251 coordinate surveys were obtained for each receiver from the conducted observations, after rejecting incomplete observations. The data obtained were aligned using the Method of Least Squares with conditional equations.

The observation results were aligned in the flat rectangular coordinate system PL-2000. The system was developed based on the mathematically unique alignment of points on the reference spheroid GRS80 with the corresponding points on the plane, in accordance with the Gauss-Krüger projection theory, and is valid in the territory of the Republic of Poland [48].

Because of the large number of data, the alignment results for only one measurement epoch (marked as 20190717_104340150), conducted in postprocessing, are presented below. The following input data were obtained from satellite observations (Table 2):

- coordinates of GNSS antennas on the MSP,
- errors in determining the GNSS antenna position coordinates.

**Figure 5:**
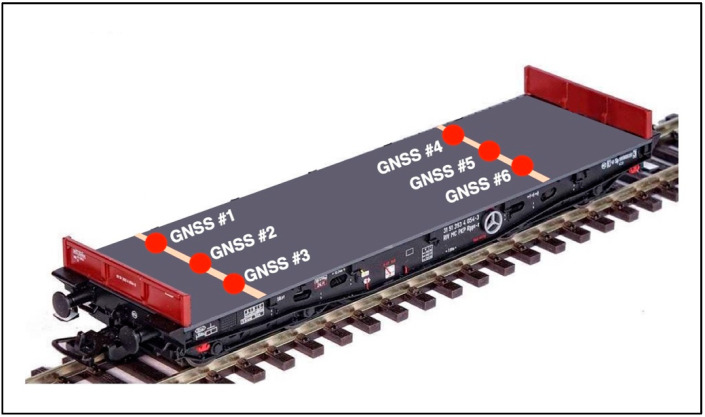
The position of GNSS receivers on the mobile surveying platform.

The position of antennas on the surveying platform is shown in the Figure 5.

Moreover, for further calculations, the measured distances between antennas (Figure 4) as well as errors of their determination: $m_{1-3}=0.0031$, $m_{1-4}=0.0033$, $m_{2-5}=0.0034$, $m_{3-6}=0.0035$, $m_{4-6}=0.0030$, resulting from the total station instrumental errors, were used. In the calculations, the measured distances to three reference stations being part of the SmartNet network [49] were used. The coordinates of the station exposures are provided in the Table 3.

The measured data allowed the system of equations comprising 18 observation equations (2) and 5 conditional equations (1) to be recorded. The solution to the alignment task (14) for the data provided above was possible on the assumption that the matrices $\overset{⌢}{A}$ and $\overset{⌢}{l}$ have the following forms:

$$\overset{⌢}{A}=\left[\begin{array}{cccccccccccc} -0.46423 & -0.88571 & 0 & 0 & 0 & 0 & 0 & 0 & 0 & 0 & 0 & 0\\ 0 & 0 & -0.46425 & -0.88570 & 0 & 0 & 0 & 0 & 0 & 0 & 0 & 0\\ 0 & 0 & 0 & 0 & -0.46427 & -0.88569 & 0 & 0 & 0 & 0 & 0 & 0\\ 0 & 0 & 0 & 0 & 0 & 0 & -0.46422 & -0.88572 & 0 & 0 & 0 & 0\\ 0 & 0 & 0 & 0 & 0 & 0 & 0 & 0 & -0.46425 & -0.88571 & 0 & 0\\ 0 & 0 & 0 & 0 & 0 & 0 & 0 & 0 & 0 & 0 & -0.46427 & -0.88570\\ 0.04475 & 0.99900 & 0 & 0 & 0 & 0 & 0 & 0 & 0 & 0 & 0 & 0\\ 0 & 0 & 0.04474 & 0.99900 & 0 & 0 & 0 & 0 & 0 & 0 & 0 & 0\\ 0 & 0 & 0 & 0 & 0.04472 & 0.99900 & 0 & 0 & 0 & 0 & 0 & 0\\ 0 & 0 & 0 & 0 & 0 & 0 & 0.04483 & 0.99899 & 0 & 0 & 0 & 0\\ 0 & 0 & 0 & 0 & 0 & 0 & 0 & 0 & 0.04481 & 0.99900 & 0 & 0\\ 0 & 0 & 0 & 0 & 0 & 0 & 0 & 0 & 0 & 0 & 0.04480 & 0.99900\\ 0.55574 & 0.83135 & 0 & 0 & 0 & 0 & 0 & 0 & 0 & 0 & 0 & 0\\ 0 & 0 & 0.55567 & 0.8314 & 0 & 0 & 0 & 0 & 0 & 0 & 0 & 0\\ 0 & 0 & 0 & 0 & 0.55560 & 0.83145 & 0 & 0 & 0 & 0 & 0 & 0\\ 0 & 0 & 0 & 0 & 0 & 0 & 0.55570 & 0.83138 & 0 & 0 & 0 & 0\\ 0 & 0 & 0 & 0 & 0 & 0 & 0 & 0 & 0.55563 & 0.83143 & 0 & 0\\ 0 & 0 & 0 & 0 & 0 & 0 & 0 & 0 & 0 & 0 & 0.55556 & 0.83148\\ 0.86346 & -0.50442 & 0 & 0 & -0.86346 & 0.50442 & 0 & 0 & 0 & 0 & 0 & 0\\ 0 & 0 & 0 & 0 & 0 & 0 & 0.86645 & 0.49927 & 0 & 0 & -0.86645 & 0.49927\\ -0.49866 & -0.86680 & 0 & 0 & 0 & 0 & 0.49866 & 0.86680 & 0 & 0 & 0 & 0\\ 0 & 0 & -0.49885 & -0.86669 & 0 & 0 & 0 & 0 & 0.49885 & 0.86669 & 0 & 0\\ 0 & 0 & 0 & 0 & -0.49815 & -0.86709 & 0 & 0 & 0 & 0 & 0.49815 & 0.86709 \end{array}\right],\;\overset{⌢}{l}=\left[\begin{array}{c} 0\\ 0\\ 0\\ 0\\ 0\\ 0\\ 0\\ 0\\ 0\\ 0\\ 0\\ 0\\ 0\\ 0\\ 0\\ 0\\ 0\\ 0\\ -0.0003749\\ -0.001608\\ 0.005840\\ 0.006241\\ 0.005869 \end{array}\right]$$

By using Equation (16), increments to the coordinates obtained from GNSS observations were obtained, which enable the determination, based on (19), of corrections to the conducted satellite observations; also, by using relationship (20), aligned coordinates of the GNSS antennas’ positions were obtained:

$$\hat{\overset{⌢}{v}}=\left[\begin{array}{r} -0.00537786\\ -0.00573547\\ -0.00466726\\ 0.00002493\\ 0.00006844\\ 0.00085569\\ 0.00015425\\ 0.00050769\\ 0.00111584\\ -0.00000250\\ -0.00000605\\ -0.00005803\\ 0.00652925\\ 0.00687617\\ 0.00541878\\ -0.00002982\\ -0.00008206\\ -0.00103008\\ -0.00000003\\ -0.00000001\\ 0.00000269\\ 0.00000019\\ 0.00000119 \end{array}\right],\;\hat{X}=\left[\begin{array}{l} 5967572.571\\ 6505456.227\\ 5967571.918\\ 6505456.609\\ 5967571.272\\ 6505456.984\\ 5967576.040\\ 6505462.280\\ 5967575.386\\ 6505462.655\\ 5967574.736\\ 6505463.031 \end{array}\right]\;$$

By using Equation (17), the antennas’ position errors of $m_{1}=0.00016$, $m_{2}=0.00077$, $m_{3}=0.00016$, $m_{4}=0.00001$, $m_{5}=0.00010$, $m_{6}=0.00003$ were determined.

A point position error is a conventional parameter that characterised the accuracy of a point’s position following the observation alignment [41]. A geometrical interpretation of the accuracy parameter is a circle with a radius of $m_{i}$. Even though the point position error is a conventional parameter and has no deeper probabilistic justification, it is very useful in many comparative analyses; therefore, Table 4 presents the percentage of error sizes, for the entire test section, along with the number of points (n) for which their values are present in the intervals (0,0.001); 〈0.001,0.005); 〈0.005,0.05); $\langle 0.05,m^{\max}\rangle$.

In analysing Table 4, it can be concluded that the error of determining the aligned coordinates of GNSS antennas does not exceed 0.005 m:

- for GNSS #1 in 99.59%
- for GNSS #2 in 99.00%
- for GNSS #3 in 99.59%
- for GNSS #4 in 99.63%
- for GNSS #5 in 99.19%
- for GNSS #6 in 99.62%

**Figure 6:**
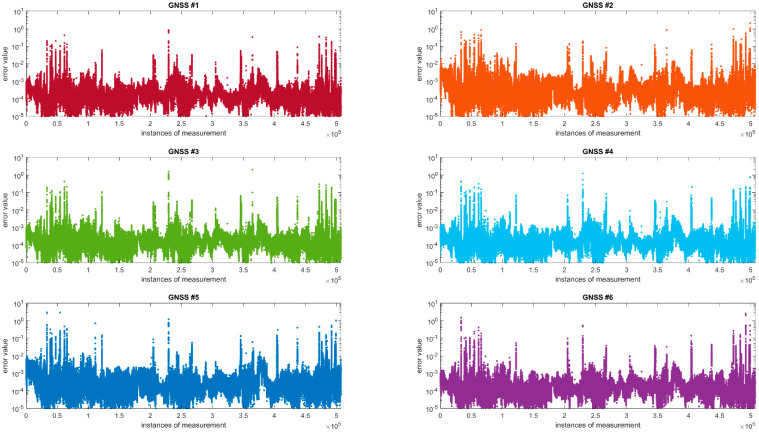
Distributions of the antennas’ position errors over the test section.

The error values in the $\langle 0.05,m^{\max}\rangle$ range appear, on average, only in 0.04%. On the other hand, Figure 6 presents the distributions of the values of errors of determining the individual receivers’ position coordinates over the entire test section.

Table 5 presents the results of statistical analyses of the series of recorded error values. The table provides minimum values, maximum values, sample mean values, confidence intervals for the mean value, variance, standard deviation and the sample size.

**Figure 7:**
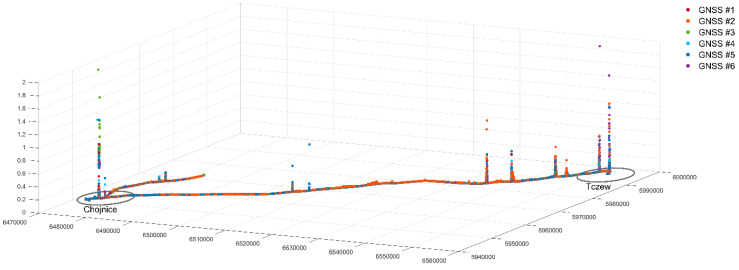
Graphical presentation of the mean error distribution over the entire test section.

The analysis was conducted independently for each of the six GNSS receivers as well as collectively for the entire series of the measured values. The total sample size amounted to 3,043,296. The mean value of the measured error values is in the order of 10^−4^. For the mean value from the sample, confidence intervals with a specified confidence level α = 0.05 were determined, which means that the mean value for the entire error population falls within the specified intervals with a probability of 0.95. It was observed that the confidence intervals were very narrow, i.e., the width of the confidence interval was smaller by two orders of magnitude than the mean value of the sample. This proves that the surveys were conducted very precisely, and the collected data set can be regarded as reliable and credible. This is also confirmed by the very low variance, in the order of 10^−5^. Despite the fact that values higher by as much as four orders of magnitude than the mean value are found in the set; these cases can be considered extremely rare and should be regarded as exceptions or anomalies. These exceptionally high values should be isolated and omitted in further research. Error values of more than 5 cm are primarily found in the urbanised area (Tczew, Chojnice), which is confirmed by Figure 7.

**Figure 8:**
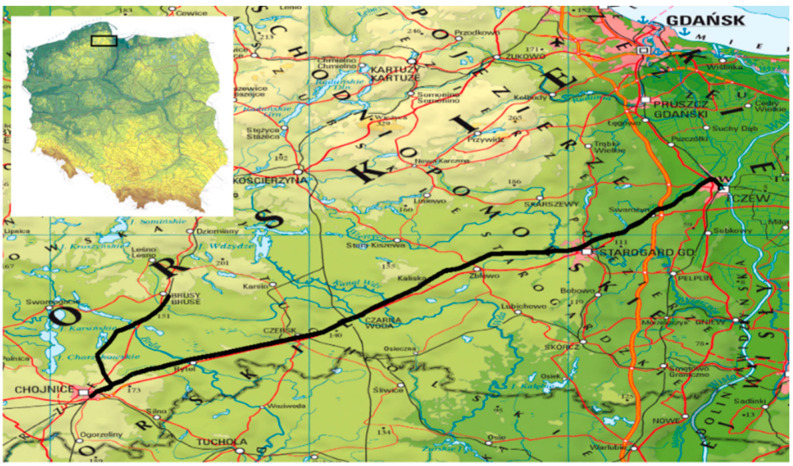
The route of railway surveys with aligned 2D coordinates.

All aligned coordinates of the position of 6 GNSS antennas (3,043,506 records in 507,251 measurement epochs) were plotted on a map of the region where surveys were performed (Figure 8).

Due to the map scale, the coordinates of GNSS antennas faithfully reproduced the route of the surveying set passage. The figure was prepared using the ArcGIS software by ESRI. After zooming in on sections of the passage route, the positions of individual GNSS receiver antennas on any given section of the route can be seen.

**Figure 9:**
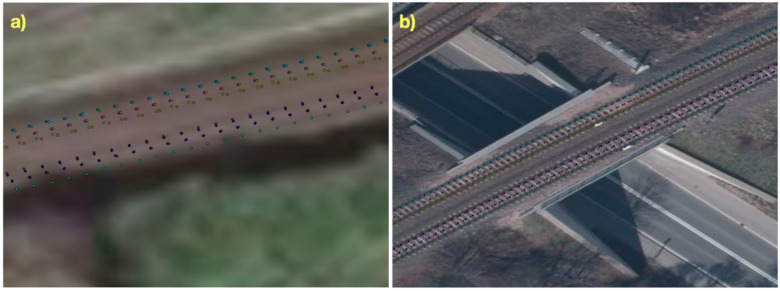
Sections of the railway route: (**a**)—near the locality of Czarna Woda, (**b**)—in an urbanised area in the town of Tczew.

For the visualisation provided in Figure 9, orthophotomaps from the GEOPORTAL website [50] were used. The images have a lowered resolution and were taken at a certain angle, which results in minor shifts being visible in the position of receivers in relation to the railway track. Moreover, it can be seen in the figure that the mobile surveying platform moved along the same route in two directions but on different tracks. It should be concluded that the conducted alignment fulfilled its purpose and yielded a very good result for such a large measurement sample implemented in a vast area characterised by various availability of satellites due to the presence of obscuring features (built-up areas, forest areas, an open area). Moreover, the mobile surveying platform moved at different speeds (30–80 km/h), which also affected the continuity of coordinate measurement by GNSS receivers.

## 5. Conclusions

The authors adapted the parametric method with conditional equations to align GNSS observations during mobile surveys. The theoretical assumptions of the method were verified using the data from GNSS satellite surveys during the inventory of the railway route of Tczew–Chojnice–Brusy–Chojnice–Tczew in northern Poland.

The 246-km-long test section is located in areas with varying satellite system signal availability (open areas, forest areas and built-up areas). This enabled the suitability of the method to be checked by aligning the observations obtained from GNSS records, which, due to the occurrence of various field-obscuring features, were characterised by different levels of accuracy and availability of GNSS signal.

The application of the surveying aligning method for satellite observations supported additionally with tacheometric surveys of the position of GNSS receiver antennas on the mobile surveying platform finally enabled the accuracy of the determinations of the antenna position coordinates to be increased and the internally consistent surveying structure to be correctly fitted within the area of measurement performance.

Analysis of the accuracy levels for the final determinations of the antennas’ position coordinates demonstrated that the parametric method with conditional equations can be applied in research tasks in which the subject of the study is the internally consistent surveying structure and the obtained observations about the positions of these structures are additionally supported with highly accurate surveys inside the structure under analysis.

The research results described in this article are the next step in checking the suitability of the LSce method in rail transport. Earlier, the authors checked its usefulness on tram tracks [48]. Moreover, the use of the mobile measurement platform is described, for example, in [18,19,20,21]. Previous studies indicate the repeatability of the results and an increase the accuracy of determinations of the position of GNSS receivers’ coordinates.

To confirm the possibility for the use of a mobile surveying platform, the authors are preparing to conduct subsequent surveying campaigns on various test sections under different conditions of GNSS signal availability. However, to confirm the universality of the proposed adaptation of the alignment method, testing should be conducted using other vehicles. It will then be possible to fully assess the suitability of the technologies applied and the methods developed for various forms of transport.

## Figures and Tables

**Table 1:** Selected parameters of a LEICA GS 18T receiver.

Parameter	Value
**Signals Tracked**	GPS (L1, L2, L2C, L5), Glonass (L1, L2, L2C, L32), BeiDou (B1, B2, B32), Galileo (E1, E5a, E5b, Alt-BOC, E62), QZSS (L1, L2C, L5, L62), NavIC L53, SBAS (WAAS, EGNOS, MSAS, GAGAN), Band L
**Initialisation Time**	Normally 4 s
**Rtk Accuracy**	A single baseline: Hz 8 mm + 1 ppm/V 15 mm + 1 ppm Network RTK: Hz 8 mm + 0.5 ppm/V 15 mm + 0.5 ppm
**Postprocessing Accuracy**	Static mode (phase), long-term observations: Hz 3 mm + 0.1 ppm/V 3.5 mm + 0.4 ppm, Static and fast static mode (phase): Hz 3 mm + 0.5 ppm/V 5 mm + 0.5 ppm
**Weight**	1.20 kg/3.50 kg—a standard configuration of an RTK receiver on a pole
**Dimensions**	173 mm × 173 mm × 108 mm
**Position Measurement**	5 Hz/20 Hz

**Table 2:** Coordinates of GNSS receiver antennas along with the errors of their determination, obtained from satellite observations.

GNSS Receiver	*x*	*y*	*m*
GNSS #1	5967572.5583	6505456.2272	0.0669
GNSS #2	5967571.9058	6505456.6093	0.2609
GNSS #3	5967571.2635	6505456.9836	0.0966
GNSS #4	5967576.0405	6505462.2802	0.0046
GNSS #5	5967575.3857	6505462.6552	0.0285
GNSS #6	5967574.7377	6505463.0309	0.0404

**Table 3:** Coordinates of the SmartNet network reference stations.

Station Name	*x*	*y*
**Starogard Gdański**	5981664.913	6532343.251
**Konarzyny**	5965574.243	6460849.453
**Czersk**	5962606.324	6498027.094

**Table 4:** Distribution of mean errors of determining the antenna positions following the alignment over the entire section of the track being measured (n = 507,251) and their percentage for the entire recording.

Receiver	m [m]								*m* ^max^
	0–0.001		0.001–0.005		0.005–0.05		0.05–max		
	n	%	n	%	n	%	n	%	
**GNSS #1**	495,231	97.63%	9938	1.96%	1965	0.39%	116	0.02%	0.831
**GNSS #2**	455,199	89.74%	46,981	9.26%	4738	0.93%	333	0.07%	2.068
**GNSS #3**	496,176	97.82%	8971	1.77%	1963	0.39%	141	0.03%	1.983
**GNSS #4**	492,935	97.18%	12,438	2.45%	1707	0.34%	171	0.03%	1.206
**GNSS #5**	465,407	91.75%	37,738	7.44%	3824	0.75%	282	0.06%	2.908
**GNSS #6**	497,205	98.02%	8122	1.60%	1784	0.35%	140	0.03%	1.506

**Table 5:** Results of statistical analyses of the error of determining the antenna position coordinates following the alignment.

Receiver	Minimal Value	Maximal Value	Mean Value	Confidence Intervals for the Mean (α = 0.05)	Variance	Standard Deviation	Sample Size
**GNSS #1**	1.592 × 10^−6^	0.831	3.099 × 10^−4^	$\langle 2.985\times 10^{-4},3.213\times 10^{-4}\rangle$	1.712 × 10^−5^	4.138 × 10^−3^	507,216
**GNSS #2**	4.557 × 10^−7^	2.068	6.161 × 10^−4^	$\langle 6.004\times 10^{-4},6.318\times 10^{-4}\rangle$	3.251 × 10^−5^	5.702 × 10^−3^	507,216
**GNSS #3**	5.999 × 10^−7^	1.983	3.297 × 10^−4^	$\langle 3.110\times 10^{-4},3.485\times 10^{-4}\rangle$	4.651 × 10^−5^	6.820 × 10^−3^	507,216
**GNSS #4**	9.948 × 10^−7^	1.206	3.047 × 10^−4^	$\langle 2.961\times 10^{-4},3.133\times 10^{-4}\rangle$	9.743 × 10^−5^	3.121 × 10^−3^	507,216
**GNSS #5**	8.365 × 10^−7^	2.908	5.643 × 10^−4^	$\langle 5.395\times 10^{-4},5.890\times 10^{-4}\rangle$	8.087 × 10^−5^	8.993 × 10^−3^	507,216
**GNSS #6**	6.732 × 10^−7^	2.613	3.611 × 10^−4^	$\langle 3.327\times 10^{-4},3.895\times 10^{-4}\rangle$	1.065 × 10^−4^	1.032 × 10^−2^	507,216
**Total**	4.557 × 10^−7^	2.908	4.143 × 10^−4^	$\langle 4.064\times 10^{-4},4.222\times 10^{-4}\rangle$	4.890 × 10^−5^	6.993 × 10^−3^	3,043,296
